# Observations on the ex situ perfusion of livers for transplantation

**DOI:** 10.1111/ajt.14687

**Published:** 2018-03-14

**Authors:** Christopher J. E. Watson, Vasilis Kosmoliaptsis, Caitlin Pley, Lucy Randle, Corinna Fear, Keziah Crick, Alexander E. Gimson, Michael Allison, Sara Upponi, Rebecca Brais, Ina Jochmans, Andrew J. Butler

**Affiliations:** ^1^ Department of Surgery University of Cambridge Addenbrooke's Hospital Cambridge UK; ^2^ NIHR Blood and Transplant Research Unit in Organ Donation and Transplantation, University of Cambridge Cambridge UK; ^3^ NIHR Cambridge Biomedical Research Centre Cambridge UK; ^4^ Department of Medicine Cambridge University Hospitals NHS Foundation Trust Cambridge UK; ^5^ Department of Radiology Cambridge University Hospitals NHS Foundation Trust Cambridge UK; ^6^ Department of Pathology Cambridge University Hospitals NHS Foundation Trust Cambridge UK; ^7^ Department of Microbiology and Immunology Laboratory of Abdominal Transplantation Katholieke Universiteit Leuven Leuven Belgium; ^8^ Department of Abdominal Transplant Surgery University Hospitals Leuven Leuven Belgium

**Keywords:** clinical research/practice, liver allograft function/dysfunction, liver transplantation/hepatology, metabolism/metabolite, organ perfusion and preservation

## Abstract

Normothermic ex situ liver perfusion might allow viability assessment of livers before transplantation. Perfusion characteristics were studied in 47 liver perfusions, of which 22 resulted in transplants. Hepatocellular damage was reflected in the perfusate transaminase concentrations, which correlated with posttransplant peak transaminase levels. Lactate clearance occurred within 3 hours in 46 of 47 perfusions, and glucose rose initially during perfusion in 44. Three livers required higher levels of bicarbonate support to maintain physiological pH, including one developing primary nonfunction. Bile production did not correlate with viability or cholangiopathy, but bile pH, measured in 16 of the 22 transplanted livers, identified three livers that developed cholangiopathy (peak pH < 7.4) from those that did not (pH > 7.5). In the 11 research livers where it could be studied, bile pH > 7.5 discriminated between the 6 livers exhibiting >50% circumferential stromal necrosis of septal bile ducts and 4 without necrosis; one liver with 25‐50% necrosis had a maximum pH 7.46. Liver viability during normothermic perfusion can be assessed using a combination of transaminase release, glucose metabolism, lactate clearance, and maintenance of acid‐base balance. Evaluation of bile pH may offer a valuable insight into bile duct integrity and risk of posttransplant ischemic cholangiopathy.

## Abstract 2

The authors describe a series of ex situ normothermic liver perfusions in which they examine biochemical markers of hepatocyte and cholangiocyte biochemistry in an effort to predict posttransplant viability.

**Abbreviations**
ALT: Alanine transaminase
AST: Aspartate transaminase
DBD: Donation after brain death
DCD: Donation after circulatory death
DRI: Donor risk index
H&E: Hematoxylin and Eosin
HA: Hepatic artery
IC: Ischemic cholangiopathy
ITBL: Ischemic type biliary lesion
DLI: United Kingdom Donor Liver Index
MRCP: Magnetic resonance cholangiopancreatography
NESLiP: Normothermic ex situ liver perfusion
PV: Portal Vein
UK: United Kingdom

## 1 INTRODUCTION

Reliable metrics to determine whether a liver is viable and safe to transplant are currently not available. Normothermic perfusion of the liver affords an opportunity for viability assessment. Although there have been case reports and small series of liver transplants following ex situ normothermic perfusion,^1^, ^2^, ^3^, ^4^, ^5^ there are as yet no validated criteria predicting liver viability.

This paper describes our observations on the biochemistry and perfusion characteristics of 47 human livers that were normothermically perfused, of which 22 were transplanted. We used readily available biochemical and physical measurements that could be analyzed during perfusion. From these studies, we have demonstrated criteria that may be helpful in determining viability and identified others that are not as discriminatory as has been suggested.^5^, ^6^

## 2 MATERIALS AND METHODS

### 2.1 Livers

Livers that had been considered unsuitable for transplantation by all UK liver centers, and high‐risk livers which we elected to assess by perfusion prior to implantation, were included. All were preserved initially with University of Wisconsin static cold storage solution.

Approvals from the local research ethics committee and the National Health Service Blood and Transplant (NHSBT) research governance were obtained for the ex situ perfusion of human livers. Transplantation of perfused livers was also approved by our institution's new interventional procedures committee. Informed consent was given by all recipients in respect of both the liver they received and the use of normothermic ex situ perfusion.

### 2.2 Normothermic ex situ liver perfusion (NESLiP)

Livers were perfused using the Liver Assist (Organ Assist, Groningen, Netherlands).^7^ The perfusate comprised leucocyte‐depleted red cells (approximately 1 litre) and either 1 litre of succinylated gelatin (Gelofusine, BBraun Medical, UK) (n = 41) or Steen solution (Xvivo Perfusion, Göteborg, Sweden) (n = 6). This was supplemented with heparin, magnesium sulphate, calcium chloride, amino acids, with sodium bicarbonate added to achieve a pH > 7.2 before NESLiP began; a 2 μg/h epoprostenol infusion ran throughout the perfusion. Bile salts were not administered. Perfusion was commenced at 20°C for all livers; the perfusate was warmed to 35‐37°C over the first 10‐30 minutes. Mean hepatic artery (HA) pressure was increased from 30 mmHg at the start of perfusion to 60 mmHg at ≥35°C; portal vein (PV) pressure was increased from 4‐6 mmHg to 8‐10 mmHg over the same period. Perfusate samples were taken at 10 and 30 minutes, and every 30 minutes thereafter. Bile was sampled when it appeared in adequate volumes for assay. Bile and perfusate gases were analyzed immediately using a Cobas b221 benchtop analyzer (Roche); other biochemistry samples were frozen and stored at −70°C until assayed. Nontransplanted livers were biopsied at hourly intervals. Vascular resistance was calculated as the perfusion pressure (mmHg) divided by the flow (L/min). Table 1 and Supplementary Figure S1 detail the perfusion protocol evolution throughout the study.

**Table 1:** Protocol evolution during study

Research livers														
Protocol variation	R1	R2	R3	R4	R5	R6	R7	R8	R9	R10 to R13	R14 to R22	R23	R24	R25
Gas delivery to oxygenator	>95% oxygen							Air with O2 supplementation if HVSaO_2_<50%						
Pre‐perfusion liver flush solution	Gelofusine							Hartmann's solution						
Perfusate colloid	Gelofusine				Steen solution			Gelofusine						
Insulin	Insulin infusion 2u/h					bolus	No insulin							
Perfusion temperature	Warmed to 37°C over 20 mins										Warmed to 35°C over 10 mins, then from 35°C to 37°C at 2 h			

Transplanted livers												
Protocol variation	T1	T2	T3	T4	T5	T6	T7	T8	T9 to T12	T13	T14 to T21	T22
Gas delivery to oxygenator	>95% oxygen						Air with O2 supplementation if HVSaO_2_<50%					
Pre‐perfusion liver flush solution	Gelofusine						Hartmann's solution					
Perfusate colloid	Gelofusine					Steen solution			Gelofusine			
Insulin	Insulin infusion 2u/h					bolus	No insulin					
Perfusion temperature	Warmed to 37°C over 20 mins									Warmed to 35°C over 10 mins, then to 37°C at 2 h		

Initial perfusions involved >95% oxygen delivery to the oxygenator, but five out of six transplanted livers on this protocol suffered vasoplegia and reperfusion syndrome so this was reduced to air,^10^ with supplementary oxygen only when the hepatic vein hemoglobin oxygen saturation (HVSaO_2_) was <50%. At the same time the protocol was changed to flush the livers with Hartmann's solution rather than Gelofusine prior to perfusion, to add a lactate load to the liver. Steen solution was used instead of Gelofusine for six perfusions. Insulin was discontinued after 12 liver perfusions since it was found not to play any part in the uptake of glucose by the liver. Reducing the perfusion temperature from 37°C to 35°C for the first 2 h was an attempt to reduce free radical production and mitigate reperfusion injury.

### 2.3 Glycogen estimation

The glycogen content of frozen right lobe liver biopsies was estimated using Zhang's adaptation of an acid hydrolysis method.^8^, ^9^ Briefly, liver biopsies were homogenized with either sodium hydroxide or hydrochloric acid. The supernatant was then mixed with Glucose Assay Reagent (Sigma Aldrich) and the glucose content measured.

### 2.4 Histology

Liver biopsies were placed in formalin and stained with hematoxylin and Eosin (H&E). Following perfusion nontransplanted livers were sliced transversely to include the major duct branches and formalin fixed. Stromal necrosis of septal bile ducts was scored on a scale of 1 to 4 after Hansen et al^10^ where Grade 1 and 2 corresponded to <25% and 25‐50% circumferential necrosis respectively and Grade 3/4 to >50% circumferential necrosis.

## 3 RESULTS

A total of 47 livers, 12 from brain dead (DBD) and 35 from circulatory death (DCD) donors, were subject to NESLiP (Table 2) between January 2014 and October 2017. Of the 28 livers accepted for possible transplantation, 20 were transplanted while 2 of the 19 livers accepted with no intent to transplant were also transplanted. Median posttransplant follow‐up is 601 days (IQR 257‐752 days).

**Table 2:** Details of the 47 livers and their perfusion characteristics

Liver number	DBD/DCD donor	Donor age	Accepted for research or possible transplant	WIT (mins)		CIT mins	US Donor Risk Index	UK Donor Liver Index	Perfusate colloid^a^	Perfusate ALT at 2 hours (iU/L)	Max rate of lactate fall^b^		Amount of NaCO_3_ added in first 4 h (mmol)	Perfusate glucose (mmol/L)		When the bile pH was maximal^c^				Cholangiopathy/histological stromal injury grade^e^	Notes
				Arrest to cold perfusion	Withdrawal to cold perfusion						mmol/L/h	mmol/L/kg/h^b^		At 2 h	At 4 h	Bile pH	Perfusate pH	Bile glucose	Perfusate glucose		
T1	DCD	57	T	10	160	360	2.01	1.98	G	1365	9.3	—	20	18.8	—	Not checked				None	
T2	DCD	24	T	5	17	419	1.92	1.45	G	1315	6.0	—	10	15.0	3.7	Not checked				None	
T3	DCD	57	T	30	41	346	2.09	1.91	G	1920	7.6	—	25	5.9	8.1	Not checked				IC	Awaiting retransplant
T4	DCD	63	T	20	31	222	2.08	2.09	G	1478	10.5	—	20	22.5	19.9	Not checked				None	
T5	DCD	45	T	17	31	445	2.12	1.69	G	3783	9.2	—	30	>40	27.0	7.56	7.17	Not checked		None	
T6	DCD	48	T	14	36	438	2.45	2.35	S	9490	4.6	—	35	9.5	8.6	Not checked				None	Primary non function
T7	DCD	55	T	11	30	396	2.51	2.03	S	1947	10.1	—	0	26.6	20.1	6.89	7.31	23.4	24.7	IC	
T8	DBD	60	T	0	0	608	2.17	1.01	S	5576	10.6	—	0	31.5	24.0	7.85	7.30	3.5	20.5	None	
T9	DCD	58	R	5	37	389	3.14	1.99	G	1118	21.5	11.6	40	38.4	30.1	7.57	7.24	7.0	22.0	None	Research offer because of traumatic injury to left lobe, mildly fatty and “long” withdrawal time
T10	DBD	39	R	0	0	618	1.47	0.96	G	418	14.0	13.3	0	8.5	6.3	7.84	7.20	1.8	14.1	None	Research offer because of poor in situ perfusion
T11	DCD	54	T	12	24	435	2.81	2.53	G	913	7.2	5.5	0	31.0	—	7.29	7.23	29.2	31.0	IC	Retransplant d188 for IC
T12	DBD	67	T	0	0	877	2.44	1.75	G	555	9.2	7.1	5	20.8	—	Insufficient bile				None	Early allograft dysfunction: INR 1.6 on day 7.
T13	DBD	71	T	0	0	245	2.00	1.15	G	4828	10.2	6.6	10	19.6	19.6	7.74	7.29	2.2	19.3	None	
T14	DCD	69	T	10	51	383	3.57	2.20	G	486	11.7	16.3	0	9.4	7.9	7.69	7.48	No reading		None	
T15	DBD	41	T	0	0	321	1.46	1.08	G	1377	10.1	6.2	5	14.4	—	7.53	7.07	No reading		None	
T16	DCD	67	T	14	24	333	3.66	2.92	G	1753	7.7	5.8	5	12.1	13.8^d^	7.31	7.25	No reading		IC	Retransplant day 107 for IC and HAT
T17	DCD	43	T	12	23	330	2.67	1.95	G	1506	6.0	4.4	15	23.3	19.4	7.92	7.17	9.1	21.9	None	
T18	DCD	66	T	13	24	448	3.17	2.24	G	2318	9.3	5.9	15	14.4	16.7^d^	7.72	7.23	2.6	15.5	None	
T19	DCD	55	T	11	33	439	2.85	2.55	G	262	11.6	15.2	10	15.9	16.4	7.66	7.25	5.6	15.7	None	
T20	DCD	29	T	10	16	292	1.69	1.43	G	216	12.6	5.6	15	18.6	17.4	7.62	7.39	2.1	18.7	None	
T21	DBD	64	T	0	0	354	2.12	1.6	G	1906	9.0	8.1	10	17.5	18.3	7.89	7.28	3.8	18.3	None	
T22	DCD	69	T	12	35	327	4.12	2.62	G	1631	5.8	4.9	10	21.6	15.7	7.72	7.27	5.8	13.4	None	
R1	DCD	52	R	10	16	539	2.21	1.77	G	3561	7.8	—	20	8.3	8.2	Not checked					
R2	DCD	34	R	13	20	913	2.71	2.02	G	4385	3.8	4.2	0	22.8	—	Not checked					
R3	DCD	39	R	16	27	523	1.69	1.39	G	8076	10.4	5.6	60	37.0	35.3	Not checked					
R4	DCD	46	R	12	420	264	1.84	1.42	G	3686	15.9	—	0	15.9	—	Not checked					
R5	DBD	55	R	0	0	600	1.70	1.19	S	2561	15.0	10.3	15	33.8	—	Not checked					
R6	DCD	64	R	10	21	384	3.60	2.26	S	740	6.7	4.1	5	24.6	18.5	Not checked					
R7	DCD	44	R	9	22	574	1.70	2.01	S	11970	10.7	5.5	0	14.7	12.2	Not checked					
R8	DCD	45	R	12	38	836	2.99	1.64	G	8348	6.8	4.5	0	10.6	7.2	Insufficient bile					
R9	DCD	76	R	12	?	611	3.14	2.38	G	1573	17.2	9.4	0	28.1	23.9	Insufficient bile					
R10	DCD	44	R	8	12	261	2.11	2.02	G	2469	3.5	2.7	30	20.6	17.2	No reading					
R11	DCD	61	T	12	18	456	2.78	2.21	G	4616	6.2	4.0	10	18.5	—	Insufficient bile				3	Not transplanted: Left lobe not perfused due to divided left accessory hepatic artery
R12	DCD	69	R	11	21	764	2.70	2.60	G	3623	16.9	13.6	0	28.0	21.4	7.72	7.42	5.4	23.7	1	Unexplained left lobe atrophy
R13	DBD	44	R	0	0	1148	1.92	0.97	G	7254	8.8	4.2	20	34.8	27.4	7.38	7.22	32.2	32.6	4	Very steatotic liver
R14	DCD	35	T	13	20	323	1.74	1.65	G	2648	12.4	8.3	0	15.8	—	7.47	7.10	6.4	14.2	4	Not transplanted due to low bile pH
R15	DCD	47	R	11	11	686	2.62	1.71	G	5386	7.5	4.8	30	26.7	24.5	Hemobilia				1	Cystic duct not ligated. Steatohepatitis on biopsy
R16	DCD	66	T	8	26	518	3.31	1.91	G	2439	8.7	4.2	40	37.6	35.9	7.40	7.33	34.5	35.9	3/4	Not transplanted: Poor glucose fall, slow lactate fall, rising potassium and high bicarbonate use, low bile pH
R17	DBD	81	R	0	0	466	2.37	1.71	G	346	12.3	11.5	0	23.4	28.8	Hemobilia				2	
R18	DCD	49	T	11	30	482	2.81	1.81	G	694	17.3	10.1	15	21.1	18.5	7.31	7.19	18.3	21.9	4	Not transplanted, low bile pH
R19	DCD	59	T	12	32	306	2.68	1.97	G	4909	10.4		10	30.1	20.9	7.44	7.21	24.7	28.2	4	Not transplanted, low bile pH
R20	DBD	55	T	0	0	328	1.79	0.99	G	4294	6.8	3.0	20	16.6	9.8^d^	7.46	7.27	4.1	8.6	2	Not transplanted, low bile pH
R21	DCD	57	R	28	?	467	3.36	2.49	G	4852	6.7	4.6	15	22.3	14.9	7.75	7.21	15.5	14.9	1	
R22	DCD	69	T	9	31	278	2.70	2.48	G	9613	10.2	6.3	10	29.0	28.1	7.61	7.21	22.2	28.1	1	Not transplanted, high ALT
R23	DCD	40	T	5	21	373	1.96	1.62	G	4424	10.8	6.2	20	32.6	29.3	Insufficient bile				3	Not transplanted, high ALT
R24	DCD	26	R	7	23	366	1.83	1.57	G	2413	9.6	3.9	25	17.6	13.8	7.75	7.15	6.1	11.9	1	2.8 kg steatotic liver, poorly perfused right lobe
R25	DCD	69	R	7	10	675	2.83	2.05	G	4088	9.0	5.0	20	31.5	27.9	7.45	7.41	30.6	27.4	4	Not transplanted, high ALT, low bile pH

The two‐hour ALT is reported above. ALT release plateaued in most livers by 2 hours and so this time point was chosen to be most representative.

? = not known.

ALT, alanine transaminase; CIT, cold ischemic time; DBD, donation after brain death; DCD, donation after circulatory death; DLI, donor liver index^34^; DRI, donor risk index^33^; HAT, hepatic artery thrombosis; INR, international normalized ratio; WIT, warm ischemic times; NaHCO_3_, sodium bicarbonate.

a G, Gelofusine; S, Steen solution.

b Maximum rate of fall of lactate between measurement points, expressed both as an absolute value and, where the liver was weighed before being perfused, as a rate per kg liver.

c At the time point when the bile pH was maximal, the pH and glucose of bile and perfusate.

d The perfusate has been supplemented with additional glucose when it fell below 10 mmol/L.

e Ischemic cholangiopathy (IC, ischemic type biliary lesions) proven on cholangiography; septal duct stromal injury graded by histological appearance, where grade 1 involves <25% circumferential stromal necrosis, 2 is 25‐50%, 3 is 50‐75%, and 4 is >75% of the circumference exhibiting necrotic stroma.^10^

### 3.1 Clinical outcomes

Of the 22 transplanted livers, the first 12 have been reported previously.^7^ One recipient died following primary nonfunction (T6), one (T12) developed early allograft dysfunction by Olthoff's criteria (day 7 INR1.6),^11^ and 4 recipients have developed ischemic‐type biliary lesions (ITBL) at 33 to 89 days posttransplant of whom 3 required retransplantation. None of the last 16 cases suffered reperfusion syndrome or vasoplegia, something that affected cases T2 to T6.^7^

### 3.2 Hepatic artery and portal vein resistance

**Figure 1:**
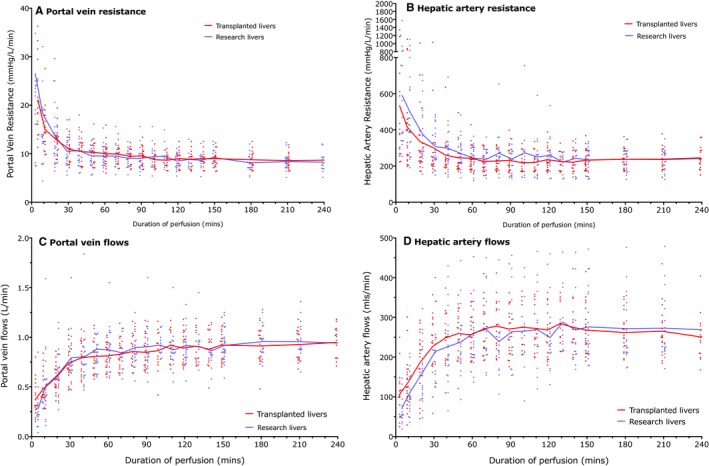
Vascular resistance and flows during perfusions. Lines represent trends in mean resistance (A and B) or flows (C and D) in portal vein (A and C) and hepatic artery (B and D); dots represent individual perfusions. Once perfusion temperature was ≥35°C, HA and PV pressures were fixed at 60 mmHg and 8 to 10 mmHg, respectively. HA and PV resistances both fall to a steady state by 60 minutes of perfusion (HA 60 median resistance 235 and 254 mmHg/L/min for transplanted and research livers respectively, and PV median 60 minute resistance 9.95 and 9.3 mmHg/L/min, respectively). Vascular flows are dependent on resistance and perfusion pressure, and at 60 minutes median HA flows were 250 and 236 mls/min for transplanted and research livers, respectively, and PV flows were 0.81 and 0.85L/min, respectively.( HA, hepatic artery; PV, portal vein

HA resistance was almost 25‐fold higher than PV resistance, with resistance being highest at the onset of perfusion and falling to reach steady state by 60 minutes; there was no difference between transplanted and nontransplanted livers (Figure 1). There was no correlation between the 60‐minute HA or PV resistance and transaminase release, a marker of hepatocellular damage.

### 3.3 Transaminase release

**Figure 2:**
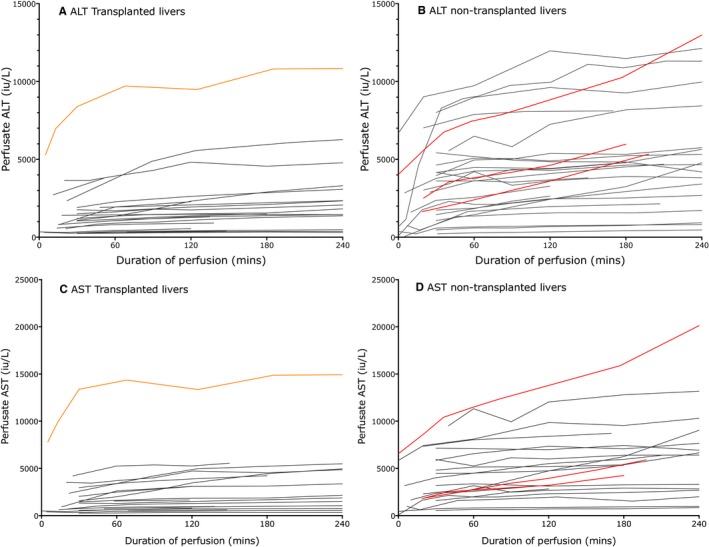
Graphs showing the change in perfusate ALT (A and B), AST (C and D) concentrations throughout the course of NESLiP of transplanted (B and D) and nontransplanted (A and C) livers. Figure 2 (A to D) shows the perfusate ALT and AST rising following the start of NESLiP. ALT and AST release from the livers had abated by two hours in most livers. In B and D, the orange line is the liver T6 that developed primary nonfunction. In A and C, three livers show continued transaminase release beyond 2 hours suggesting ongoing hepatocellular damage; from highest to lowest peak ALT and AST, these were livers R8, R11, and R1. ALT, alanine transaminasel; AST, aspartate transaminase; NESLiP, normothermic ex situ liver perfusion

Alanine and aspartate transaminase (ALT and AST, respectively) concentrations in the perfusate plateaued by 2 hours in most livers; the 2‐hour ALT was therefore taken to be representative of the degree of hepatocellular damage (Figure 2). ALT and AST were closely correlated (*r* = .94, *P* < .001).

**Figure 3:**
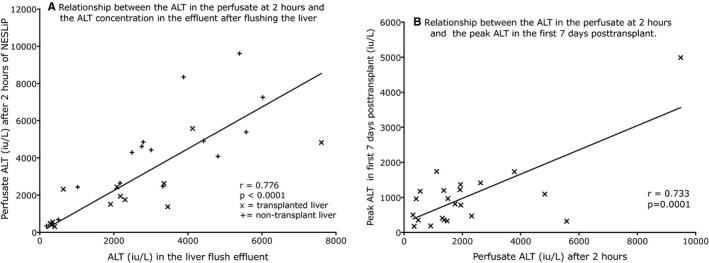
Relationship between the ALT in the perfusate at 2 hours and (A) the ALT concentration in the preservation effluent and (B) the peak ALT in the first 7 days posttransplant. (A) 28 livers were flushed with Hartmann's solution before undergoing NESLiP from which transaminases were measured. There is a strong correlation between the concentration of ALT in the effluent Hartmann's solution after it has flushed through the liver and the perfusate ALT at 2 hours (Pearson *r* = .776, *P* < .001, linear regression equation *y* *= 1.122x*); the symbol x indicates transplanted livers while + indicates a liver that was not transplanted. (B) There is a correlation between the ALT after 2 hours of NESLiP and the peak ALT in the first 7 days posttransplant (Pearson *r* = .733, *P* = .0001, linear regression equation, *y = 0.35x+280*). ALT, alanine transaminase; NESLiP, normothermic ex situ liver perfusion

ALT concentrations were measured in the effluent of 28 livers after flushing with compound sodium lactate (Hartmann's) solution prior to NESLiP. The effluent ALT was correlated with the ALT of the perfusate 2 hours after starting NESLiP (Figure 3A, r = .78, *P* < .0001; AST *r* = .649, *P* = .031). Similar data were obtained for AST (not shown). There was a correlation between the two‐hour perfusate ALT and the peak ALT in the first seven days posttransplant for the 22 transplanted livers (Figure 3B, r = .73, *P* = .0001). In the 12 transplanted livers where effluent ALT had been measured there was no correlation with the peak ALT posttransplant (*r* = .18, *P* = .58).

### 3.4 Lactate concentration

**Figure 4:**
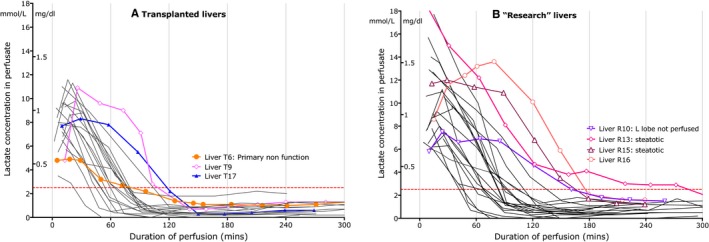
Lactate concentrations for livers at the start of NESLiP. The lactate concentration in the perfusate fell to below 2.5 mmol/L (dotted red line) in all but five livers by 2 hours, and all but one liver by 3 hours. Liver R10 was a DCD donor with a nonperfused replaced left hepatic artery and consequently a poorly perfused left lobe, which was presumably producing lactate while the well perfused right lobe was metabolising it. R13 and R15 were markedly steatotic livers. Liver T6 is highlighted in orange and was the only liver to suffer primary nonfunction following transplantation. Liver T9 had an intraparenchymal contusion of the left lobe following trauma. Supplementary Figure S1 shows the absolute change in lactate over time for each liver. NESLiP, normothermic ex situ liver perfusion

The baseline lactate concentration varied and was higher in livers flushed with Hartmann's solution (Figure 4). Fifteen livers (32%) exhibited an initial rise in lactate within the first 30 minutes of NESLiP before falling. The concentration fell to below 2.5 mmol/L by 180 minutes in all but one (2%) liver. The peak rate of fall of lactate varied between livers from 3.5 to 21.5 mmol/L/h (2.7 to 16.3 mmol/L/kg/h, Supplementary Figure S2); there was no correlation between this and ALT or AST (data not shown).

### 3.5 Glucose metabolism

**Figure 5:**
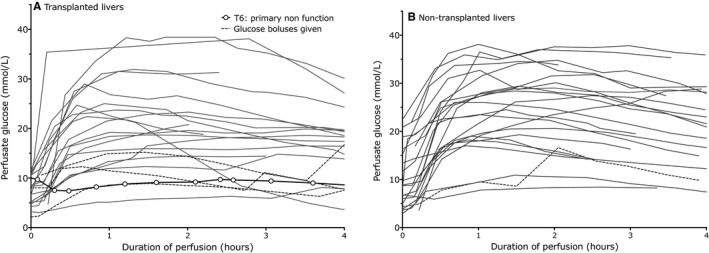
Glucose concentrations during the course of normothermic perfusion in (A) transplanted livers and (B) nontransplanted livers. Perfusate glucose concentrations rose for the first 1 to 2 hours during NESLiP, and then in all but one perfusions glucose fell at varying rates but each suggestive of zero order kinetics. Dashed lines represent livers where the initial perfusate glucose concentration was low and glucose boluses were given to probe liver function; the heavy line with open circle symbols in (A) is the glucose profile for the liver that suffered primary nonfunction. NESLiP, normothermic ex situ liver perfusion

In spite of the absence of exogenous glucose in the perfusate, the perfusate glucose concentration rose above 10 mmol/L in all but five livers, and exceeded 40 mmol/L in one case (Figure 5). Where the glucose subsequently fell, the peak was reached by 2 hours. The rate of fall varied between 0.67 and 3.8 mmol/L/kg/h, appeared to follow zero order kinetics, and was independent of insulin, whether given as continuous infusion or as boluses of up 150units (data not shown). Insulin was therefore omitted from the last 35 NESLiPs.

#### 3.5.1 Glycogenolysis

To investigate whether the glucose rise could be attributed to glycogenolysis, glycogen estimations were made on wedge biopsies of four livers (Supplementary Figure S3). Glycogen content ranged from 3.8 to 47.1 mg/g liver at the start of NESLiP, falling initially during perfusion before increasing as the perfusate glucose concentration fell.

#### 3.5.2 Preservation solution

Twenty‐seven livers that had been cold stored in UW solution for a median 435 mins (range 222 to 1148) were flushed with a liter of Hartmann's solution prior to NESLiP. The glucose concentration of the resulting effluent ranged from 2.7 to 22.1 mmol/L (1.2 to 12.6 mmol/L/kg), and was proportional to the peak glucose during NESLiP (Supplementary Figure 4, r = .72, *P* < .001), suggesting that the rate of glycogenolysis was also proportional. Neither effluent glucose nor peak glucose were strongly related to cold ischemic time prior to NESLiP (*r* = −.11, *P* = .57 for effluent; *r* = .38, *P* = .04 for peak glucose). Effluent glucose showed a trend to being higher in DCD livers than DBD livers (DBD median 9.7, IQR 5.5‐11.2; median DCD 12.2, IQR 6.8‐22.1 mmol/L).

### 3.6 Glucose challenge to interrogate a non‐rising perfusate glucose

**Figure 6:**
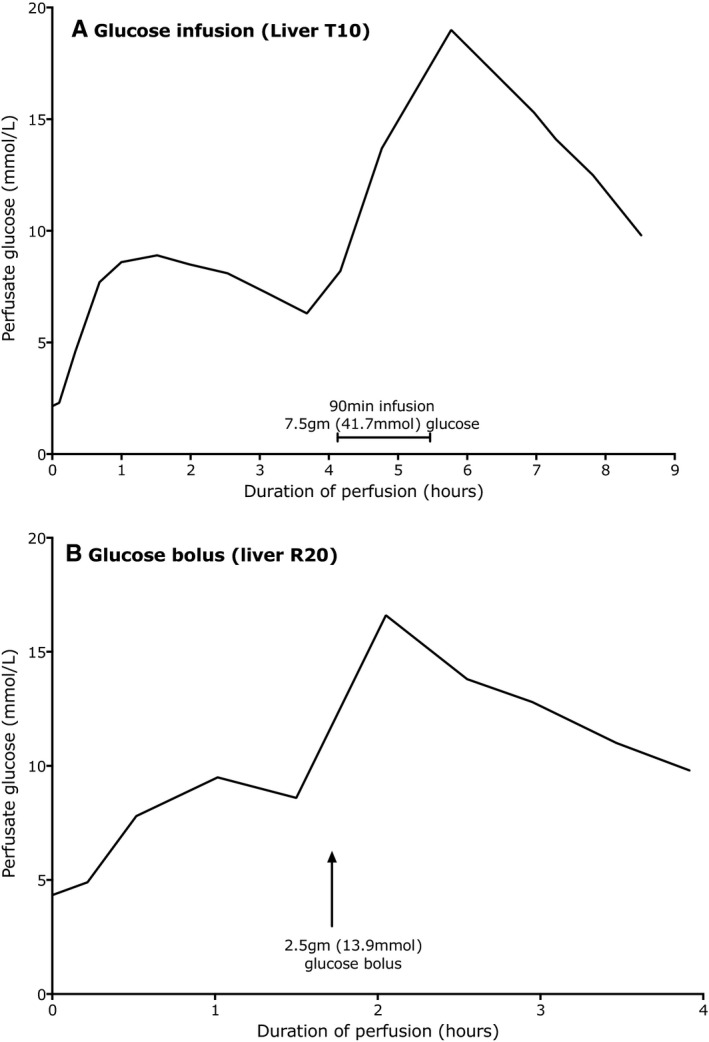
Response of two livers with an initially “low” perfusate glucose to a glucose challenge to confirm functional capacity. (A) shows the glucose concentration in the perfusate for liver T10. The glucose only rose to 8.9 mmol/L by 90 minutes. A glucose infusion was begun at 190 minutes, delivering 7.5 g (41.6 mmol), following which glucose fell. (B) shows the glucose profile of liver R20. After 90 minutes, during which a peak glucose of 9.5 mmol/L was achieved, a bolus of 2.5 g (13.9 mmol) was added. The glucose rose to 16.6 mmol/L and fell exhibiting zero order kinetics

Two of the four livers which did not generate a perfusate glucose above 10 mmol/L were interrogated with additional glucose (Figure 6). Liver T10 had a 7.5 g infusion over 90 minutes, following which the glucose was seen to fall, and liver R23 was given a 2.5 g glucose bolus, again with a fall.

### 3.7 Maintenance of perfusate pH

The perfusion protocol required bicarbonate administration to maintain a pH ≥ 7.2 in the HA perfusate, hence bicarbonate replacement is a useful surrogate of the liver's ability to regulate pH (Supplementary Figure 7). The median bicarbonate requirement was 10 mmol (range 0 to 60, IQR 0 to 20), with most supplementation occurring during the first two hours. Three livers required more than 30 mmol of bicarbonate, one of which (T6) was transplanted and suffered primary nonfunction.

### 3.8 Bile flow

**Figure 7:**
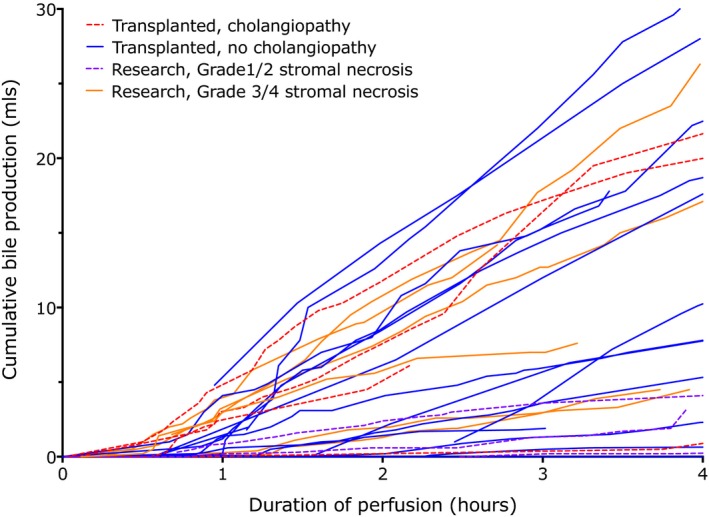
Bile flow during normothermic perfusion. Livers produced bile at a variable rate and the rate did not correlate with bile duct damage or posttransplant outcome. Each line represents a single liver perfusion, colored according to whether they were transplanted and developed cholangiopathy or not, or for research livers, whether there was grade 3 or 4 stromal necrosis of the major septal ducts on histology or not. Research livers early in the program did not have detailed histology on major septal ducts and are omitted

Recordable bile production began between 30 and 150 minutes after the start of perfusion, with the delay being in part attributable to dead space in the biliary catheter (Figure 7). Bile production varied from 0 to 60 mL after four hours. There was no relation between the rate of bile production and posttransplant function, incidence of cholangiopathy in transplanted livers or degree of stromal necrosis in nontransplanted livers.

### 3.9 Bile duct histology

**Figure 8:**
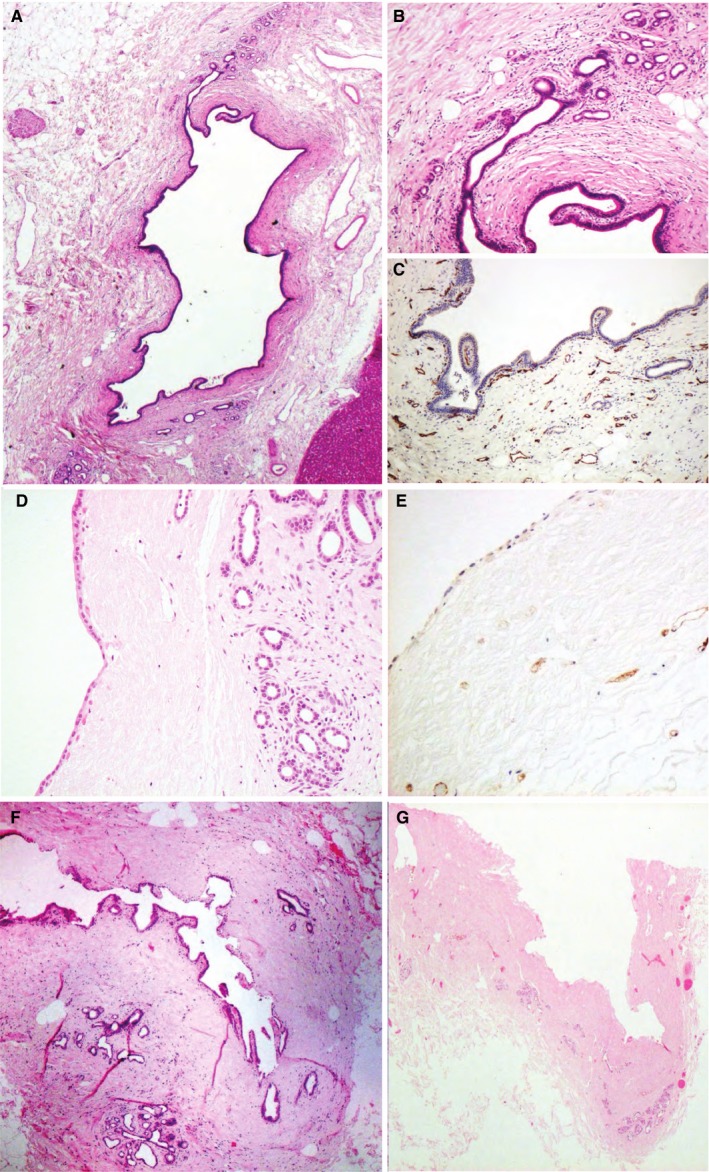
Bile duct histology illustrating normal intrahepatic ducts and ducts with moderate/severe (grade 3/4) stromal necrosis. (A) is a normal septal duct (H&E ×20) from liver R24 showing preserved epithelium, intact peribiliary glands ([B] H&E ×100), and endothelial staining in the viable stroma ([C], CD31 stain, ×100). This is grade 1 stromal necrosis (<25% of circumference affected). (D) and (E) are from liver R22; (D) (H&E ×100) shows an intact epithelial layer with preservation of some peribiliary glands but an acellular necrotic stroma indicating widespread necrosis; (E) (CD31 stain, 100x) shows relatively little CD31 staining signifying loss of endothelial cells within the stroma. (F) is from liver R14 (H&E ×40) showing patchy loss of surface epithelium, preservation of some peribiliary glands and stromal necrosis affecting between 50% and 75% of the circumference of the duct (Grade 3). (G) is from liver R21 (H&E ×40) showing loss of surface epithelium, degenerate peribiliary glands and complete stromal necrosis in an effectively dead duct (Grade 4). H&E, hematoxylin and eosin

Contrary to previous reports,^12^ common hepatic duct histology was not informative with loss of superficial epithelium being common, presumably due to handling and cannulation artefacts. Therefore, the technique of preparing research livers postperfusion was changed to produce transverse sections through the hilum which included septal ducts; 15 research livers were treated in this way (Figure 8).

The peri‐biliary vascular plexus was rarely seen on H&E, and neither was hemorrhage; the perfusate used leucocyte‐depleted erythrocytes so ischemia‐related inflammation could not be graded by leucocyte infiltration, and when present leucocytes suggested infiltration in the donor prior to retrieval. Vascular endothelium was identified using a CD31 specific stain, and loss of capillary CD31‐staining corresponded to the areas of stromal necrosis. Eight livers showed grade 3 or 4 stromal necrosis, two grade 2 (one of which produced no bile), and five grade 1 (minimal) stromal necrosis; Figure 8 illustrates normal intrahepatic ducts and those with severe ischemic damage.

### 3.10 Bile chemistry

**Figure 9:**
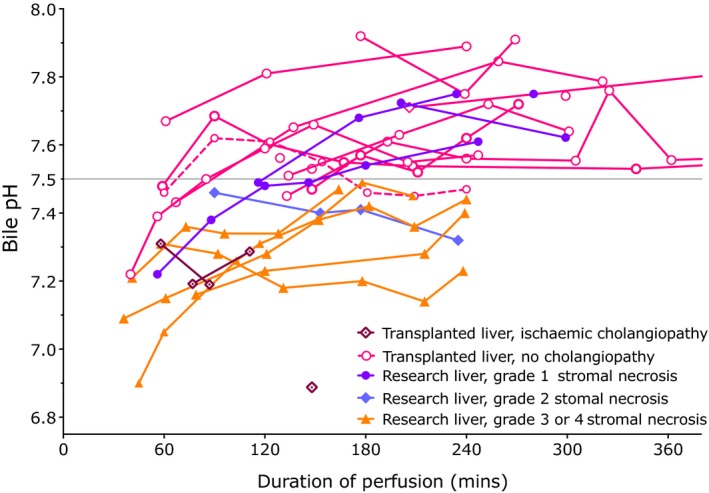
Change in pH of bile during perfusion. Figure illustrating the change in biliary pH over time in NESLiP livers. Transplanted livers which did not develop cholangiopathy are indicated in pink, those that did are in mauve. Also shown are the biliary pH measurements of livers not transplanted. These are colored such that those with minimal evidence of stromal necrosis of major septal bile ducts (grade 1) on histology are in purple, grade 2 (25‐50% circumferential necrosis) in blue, and those with greater degrees of stromal necrosis, typically affecting >50% of the circumference, are in orange. pH appears to rise initially after the start of perfusion, and then plateau above pH 7.5 in livers with viable bile ducts with the exception of liver T20 in which the pH fell back to under 7.5 (dashed pink line). )NESLiP, normothermic ex situ liver perfusion

Bile was analyzed at intervals during perfusion, the frequency of measurement depending on the amount of bile produced. Figure 9 shows the bile pH during perfusion. Of the 16 transplanted livers for which biliary pH was measured, the 3 that were unable to produce bile with a pH > 7.4 (livers T7, T11, and T16) developed cholangiopathy. The other transplanted livers all achieved a biliary pH > 7.5, often following an initial lower pH at the start of perfusion. Of the 11 research livers for which biliary pH was measured, four achieved a pH > 7.5 and these had minimal stromal necrosis of the major intrahepatic ducts (grade 1) on histological examination. Seven of the remaining eight livers had a bile pH < 7.5, most <7.4 and histology revealed moderate or severe stromal necrosis (grade 3 or 4) in addition to epithelial loss and loss of peribiliary glands.

**Figure 10:**
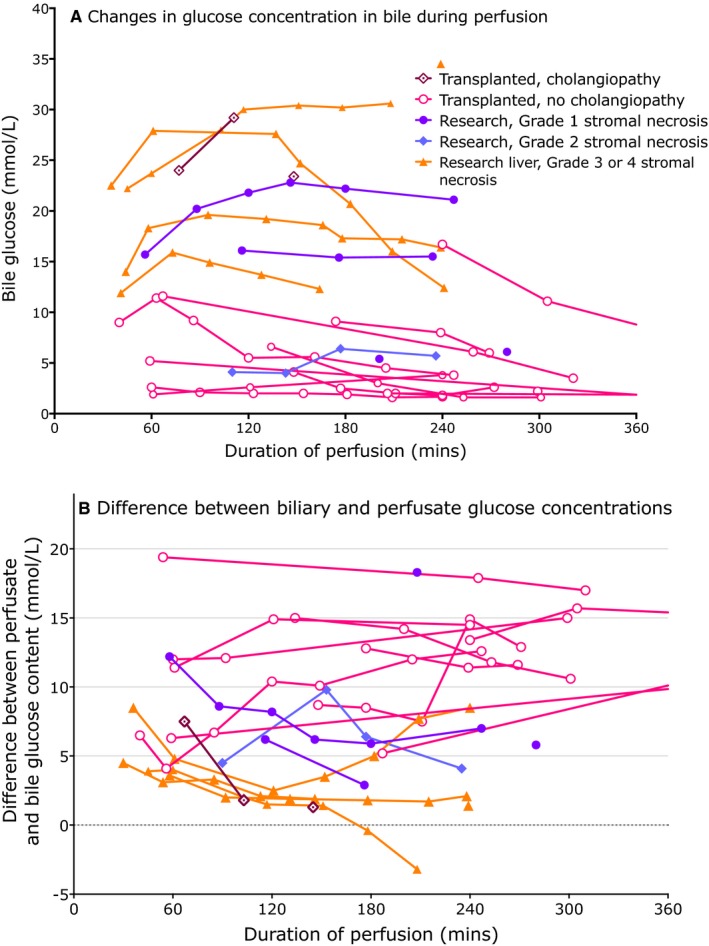
(A) The glucose concentration in bile during perfusion and (B) the difference between biliary glucose and perfusate glucose concentrations. (A) Glucose is reabsorbed from bile by cholangiocytes, so normal bile has a low glucose. The presence of glucose was associated with cholangiopathy or duct injury. Since perfusate glucose concentrations were often high, impaired cholangiocyte function often resulted in a very high glucose reflecting the high levels in the perfusate at the time. (B) depicts the difference between perfusate and bile glucose showing that most healthy ducts exhibited a large glucose gradient while diseased ducts did not

**Figure 11:**
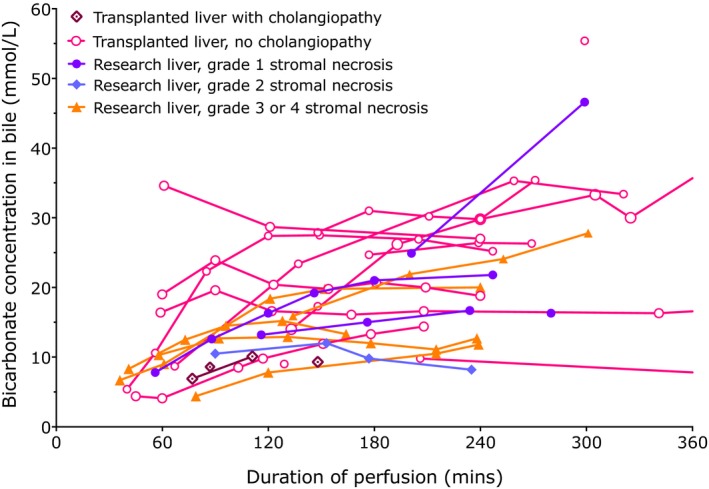
Biliary bicarbonate concentration. The bicarbonate concentration in bile increases early after perfusion as bile starts to be produced, but appears to be less discriminatory than the bile pH

Bicarbonate, which is actively secreted by cholangiocytes,^13^ and glucose, which is actively resorbed by functioning cholangiocytes,^14^ were also measured in bile and related to the perfusate concentrations. Ducts which went on to develop cholangiopathy, and those with grade 3/4 stromal necrosis were associated with lower bile bicarbonate and a bile glucose content similar to perfusate, while ducts that had good histological appearance or did not develop cholangiopathy had a lower glucose (Figures 10 and 11).

## 4 DISCUSSION

Validated guidelines to assess liver function and viability during normothermic ex situ liver perfusion do not exist. The available literature hints at HA and PV flow, lactate clearance,^5^ and bile production,^6^, ^15^ with the only clinically based evidence being derived from six human liver perfusions.^5^ Our observations, made on 47 livers declined for transplantation or which we considered to need pre‐implant assessment, document the behavior of less‐than‐ideal livers subject to NESLiP and identifies parameters that might be valuable in assessing livers at the margins of acceptability.

HA and PV flow are dependent on perfusion pressure and resistance within the vascular bed, the latter being the property intrinsic to the liver. We show that resistance in HA and PV falls in the first 60 minutes of perfusion before a steady state is reached. The resistances did not appear to correlate with outcome or biochemical markers of hepatocellular injury. Resistance is commonly used as a discriminator in hypothermic kidney perfusion, although evidence for its predictive utility is poor.^16^, ^17^ Similarly there is evidence in hypothermic liver perfusion that resistance does not reflect warm ischemic injury.^18^

Lactate clearance is used clinically as a marker of immediate function post–liver engraftment, and it is understandable that it has been attractive in the assessment of livers undergoing NESLiP. However, the massive capacity of a liver coupled with the small volume of the perfusion circuit (2 litres in our case) means that lactate clearance can occur with little viable hepatocellular function. Indeed, in our entire cohort of 47 high‐risk livers, only one failed to clear lactate below the 2.5 mmol/L 3‐hour threshold suggested by Mergental et al^5^ while the liver that suffered primary nonfunction reached this threshold within 90 minutes. The rate of lactate fall per unit liver weight may be a more appropriate measure of hepatocellular function. In addition, lactate concentration may be deceptively high in the presence of trauma or lobar ischemia,^7^ where lactate may be being produced by the ischemic liver segments. Hence, while a fall in lactate does not predict a viable liver, livers that were not able to clear lactate rapidly (T6, R10, Figure 4) were associated with parenchymal injury. Pre‐loading the circuit with lactate either by using a lactate containing fluid to washout the liver or using unwashed red cells, improves the utility of this test allowing rate of fall to be better assessed.

We have confirmed previous observations that the glycogen content of the liver falls during the initial perfusion period, and is replenished in the later stages of NESLiP,^19^, ^20^ suggesting that most of the glucose rise seen during perfusion originates from glycogenolysis. This process probably also explains the high glucose concentrations seen in cold storage fluid (Supplementary Figure S4), necessary for anerobic ATP generation. Glycogenolysis is known to be stimulated by glucagon, epinephrine and other stress hormones,^21^ so may start at the time of treatment withdrawal in DCD donors, and coning in DBD donors, times characterized by high levels of such hormones; it is noteworthy that glucose levels were higher in DCD donor livers. Lactate is metabolized into glucose in an oxygen‐dependent process and this will contribute in part to the raised perfusate glucose observed at the start of NESLiP.^22^, ^23^

The subsequent spontaneous fall in glucose during NESLiP probably in large part represented glucose entering the liver via the insulin‐independent GLUT2 bidirectional transporter, although some may be entering circulating erythrocytes via the GLUT1 transporter.^24^ As the glucose enters the liver it is probably incorporated into glycogen, initially in zone 3.^20^ The role of insulin in NESLiP is uncertain, since it is not necessary for glucose entry into the liver but may be important for other metabolic processes although its absence does not appear to have been deleterious in our perfusions. Insulin is known to inhibit glycogenolysis and stimulate glycogenesis, but high glucose concentrations may be a stronger stimulant at the levels seen during most perfusions, and we have demonstrated glycogen synthesis in the absence of exogenous insulin (Supplementary Figure S3). In our initial experiments we included an insulin infusion, and later a larger insulin bolus, but neither had an effect on glucose metabolism so insulin was thereafter omitted from the protocol. Where glucose levels did not rise during perfusion there are two possibilities, one being that there has been severe hepatocellular damage and the other that there is minimal ongoing glycogenolysis, possibly due to glycogen exhaustion. A low perfusate glucose was seen in the liver that developed primary nonfunction. We subsequently demonstrated that interrogation with a glucose challenge can demonstrate metabolic activity, and commend this as a simple test.

In this paper, the perfusate transaminase concentrations relate to the 2‐liter circuit, and most liver perfusate concentrations had reached a steady state by 2 hours, implying no ongoing damage. Although we do not have sufficient data to support a transaminase threshold, we have successfully transplanted a liver with a perfusate ALT of 5576iU/L (AST 4963) at 2 hours, while one with an ALT of 9490iU/L (AST 13349) suffered primary nonfunction. All but 6 of the 47 livers undergoing normothermic perfusion had an ALT <6000iu/L at 2 hours. We preferred to use ALT as a marker of hepatocellular damage rather than AST, since AST may also arise from hemolysis on the circuit.

We have shown that there is considerable transaminase release prior to NESLiP, with effluent levels correlating closely with the levels observed during perfusion. Measurement of effluent transaminase concentrations may be a useful test to perform before transplantation whenever concern exists about a liver, and may be useful to indicate those livers where further assessment and manipulation during normothermic perfusion maybe helpful. High concentrations of transaminase washing out of the liver before NESLiP suggest that significant hepatocellular damage occurred as a consequence of events during retrieval and cold storage, rather than due to reperfusion per se.^25^ We also suggest that future trials of NESLiP record effluent transaminase as an aid to interpreting the relative contribution of NESLiP to posttransplant transaminase levels, a common endpoint in such trials.^4^

The liver regulates acid–base balance by the differential metabolism of glutamine along the hepatic lobule, and requires integrity of both zone 1 hepatocytes and the peri‐central glutamine synthetase containing hepatocytes.^26^, ^27^, ^28^ Three livers undergoing NESLiP were unable to regulate pH without significant or continued requirement for bicarbonate supplementation, one of which was transplanted and suffered primary nonfunction. We chose pH ≥ 7.2 as the target for our perfusions. The liver receives blood from HA, typical pH 7.4, but also from PV, typical pH 7.2 (personal observation). HA oxygenation is also greater than PV clinically, as it is in our perfusions. This is not the case in other commercial machines, so the extent to which our observations may apply on other equipment, with different perfusion conditions, remains to be seen.

We found no evidence to support the notion that bile production affected outcome following transplantation. One liver that suffered primary nonfunction following transplant had an intermediate bile production, and livers that developed cholangiopathy were among the highest and lowest bile producers. In contrast to quantity, the pH of bile appears to provide a marker of biliary integrity. Our experience in a limited number of perfusions suggests that livers which are able to produce bile with a pH > 7.5 in the first 4 hours of perfusion do not exhibit histological features of extensive stromal necrosis of the major septal ducts or suffer ischemic‐type biliary lesions when transplanted; the converse applies when the pH ≤ 7.4. Necrosis of the stroma of the larger septal ducts was taken as a histological marker of irreversible bile duct damage in this study, and its presence was highlighted by the absence of capillaries staining with CD31.

Bile should normally contain little glucose, since while canalicular bile has a concentration similar to plasma, cholangiocytes reabsorb glucose from bile resulting in a low glucose,^14^ and this provided an additional marker of bile duct viability. A bile pH < 7.4 and glucose <10 mmol/L lower than perfusate glucose suggested significant stromal injury. Although it is possible that low perfusate pH may affect the ability to generate a pH > 7.5, this did not appear to be the case although high glucose gradients did pose more of a challenge to cholangiocyte reabsorption. Larger studies will be needed to validate the predictive role of biliary pH and glucose, and remove any bias in our observations.

Absence of a commercial source of clinical grade bile salts meant we were not able to include them in our protocol. The use of bile salts will affect bile production and may affect its pH and the incidence of cholangiopathy, but initial reports from a multicenter trial where they were used suggest cholangiopathy also occurs even when taurocholate is present (David Nasralla, personal communication). Perfusions in excess of 8 hours in the absence of bile salts have been shown to result in biliary damage in pigs,^29^ hence we limited our perfusions to under 8 hours.

The incidence of cholangiopathy in transplanted livers (4/22 = 18%) and grade 3 or 4 septal duct stromal necrosis in nontransplanted livers (8/15 = 53%) suggests that NESLiP, per se, does not prevent biliary damage. This may be because the damage relates to events prior to commencement of NESLiP. However, cholangiopathy has not been reported in DCD livers undergoing hypothermic oxygenated perfusion (HOPE).^30^ It is possible that this difference relates to biliary susceptibility to reperfusion injury by reactive oxygen species, and its mitigation by slow introduction of oxygen that characterises HOPE;^31^, ^32^ equally, the high resistance and consequent low initial arterial flows that characterise normothermic reperfusion may result in further warm ischemic injury to the ducts. The etiology of biliary injury during NESLiP need further evaluation, and will be aided by our identification of biochemical markers of cholangiocyte damage.

Other biochemical observations, not highlighted, may also provide corroborative evidence of function and damage. Urea rose during perfusion signifying metabolic activity. Perfusate potassium rose initially in all perfusions, and continued to rise where the transaminase release was high but fell in the livers exhibiting less damage and better metabolic activity (data not shown).

As this is an observational study, where the perfusion protocol evolved over time as experience accrued, there are limitations that need to be kept in mind. The significance of lower perfusate oxygenation on the incidence of vasoplegia and post reperfusion syndrome has been discussed elsewhere.^7^ We evaluated Steen solution as a replacement for Gelofusine in six perfusions but it was more expensive and the results were not superior. Recognition of a possible association of biliary pH with bile duct damage resulted in a number of livers being declined due to a low bile pH and high bile glucose. Table 3 shows the criteria we currently use to determine whether or not the liver is safe to transplant, but we fully acknowledge that early in our experience we turned down livers that we would now consider viable. Similarly, Table 3 is not meant to represent definitive criteria, but rather illustrates our current “comfort zone.”

**Table 3:** Variables associated with successful transplantation of a normothermically perfused liver in this series

Maximum bile pH > 7.5 Bile glucose concentration ≤3 mmol/L or ≥10 mmol less than perfusate glucose Able to maintain perfusate pH > 7.2 without >30 mmol bicarbonate supplementation Falling glucose beyond 2 h or perfusate glucose under 10 mmol/L which, on challenge with 2.5 g glucose, does subsequently fall Peak lactate fall ≥4.4 mmol/L/kg/h ALT <6000iU/L at 2 h

These criteria relate to livers perfused using the machine and protocol described in this study and may not be transferable to other machines which have, for example, different perfusates, perfusate volumes, or gas delivery to oxygenators. Livers outside these criteria may be viable, but we have no such evidence at present. Both cholangiocyte and hepatocyte markers of function/damage should be considered when evaluating whether a liver is suitable for transplantation. Inspection should confirm that both lobes are well perfused.

In summary, this report of the 47 normothermic perfusions we have undertaken has identified patterns of biochemical response to ischemia and reperfusion. The redundancy of metabolic activity in a liver perfused on a small circuit, together with its remarkable regenerative capacity which permits significant hepatocellular injury to be tolerated, confound attempts to define thresholds. Nevertheless, we do believe that we may have demonstrated a simple marker of biliary integrity, as well as casting doubt on the significance of bile production and thresholds of portal and hepatic artery flows as absolute markers of viability.

## DISCLOSURE

The authors of this manuscript have conflicts of interest to disclose as described by the *American Journal of Transplantation*. Lucy Randle now works for OrganOx, who make the *Metra* normothermic liver perfusion device; Andrew Butler is part owner of a patent on the circuit used by the OrganOx *Metra* device. The other authors of this manuscript have no conflicts of interest to disclose.
