# Supplementary material for: Observations on the ex situ perfusion of livers for transplantation
Source: Am J Transplant. 2018 Mar 14;18(8):2005–20. doi: 10.1111/ajt.14687 (PMC6099221; doi:10.1111/ajt.14687)
Supplement: Supplementary file 1 [file AJT-18-2005-s001.docx]

**Supplementary figure 1. Flow diagram of liver perfusion protocols**

**Supplementary figure 2. Change in lactates following start of NESLiP.**

1. **Transplanted livers: absolute lactate change from baseline**

1. **Research livers, absolute change in lactate from baseline**

1. **Change in lactate per kg liver weight for transplanted livers**

1. **Change in lactate per kg liver weight for research livers**

Note: Pre-perfusion weights were not available for early livers.

**Supplementary figure 3: Relationship between perfusate glucose and lactate, and liver glycogen content**

Above are a selection of livers showing the changes in glucose and lactate in the perfusate, and glycogen content of liver. Glycogen was assayed from wedge biopsies snap frozen at varying time points during perfusion. Baseline liver glycogen content was lowest in the 3 DCD donor livers (R7, R10, R14). Glycogen content fell in all livers, and was replenished as perfusion continued.

**Supplementary figure 4:**  **Relationship between glucose concentration in the effluent following washing a litre of Hartmann’s solution through the liver and the peak glucose during NESLiP.**

There is a correlation between the concentration of glucose in the effluent solution and the peak glucose during NESLiP (Pearson r=0.72, p<0.0001,). The plot includes a linear regression line, equation *y=1.1x + 11.4.*

**Supplementary figure 5. Cumulative bicarbonate requirement during perfusion.**

Bicarbonate replacement was given to maintain a pH≥7.2 in the HA perfusate, and hence the amount of bicarbonate reflected the liver’s ability to regulate pH. Most livers required no more than 30mmol bicarbonate during perfusion, and most replacement took place in the first 120 minutes. Three livers stand out. Liver T6 suffered primary non function, and can be seen to require continued supplementation. Liver R3 was from a donor dying from cerebral hypoxia secondary to hanging, who had an ALT of 1400 on admission, and 284 at the time of retrieval; histology showed minimal necrosis but extensive neutrophil infiltration, suggesting pre-existing injury since the perfusate was leucocyte-depleted.
